# Anti-Glomerular Basement Membrane Disease as a Potential Complication of COVID-19: A Case Report and Review of Literature

**DOI:** 10.7759/cureus.12089

**Published:** 2020-12-15

**Authors:** Sarah Nahhal, Ahmad Halawi, Hadil Basma, Ali Jibai, Zeinab Ajami

**Affiliations:** 1 Internal Medicine, Lebanese University Faculty of Medicine, Beirut, LBN; 2 Nephrology, Bahman Hospital, Beirut, LBN; 3 Nephrology, Lebanese University Faculty of Medicine, Beirut, LBN

**Keywords:** anti-glomerular basement membrane disease, goodpasture's syndrome, coronavirus, covid-19, case report

## Abstract

Since the COVID-19 outbreak has started, many reports showed that COVID-19 does not affect only the respiratory system but can alter multiple organs including kidneys. Anti-glomerular basement membrane disease (anti-GBM) is a systemic disease affecting mainly kidneys and lungs. It can sometimes be triggered by a respiratory infection such as influenza however the mechanism is not clear yet. We describe a novel case of Anti-GBM disease possibly complicating COVID-19. We report a case of a 63-year-old man who was admitted to our hospital for fever and myalgia and was found to have COVID-19. During hospitalization, he developed kidney injury along with pulmonary hemorrhage and was found to have anti-GBM antibodies. Our patient was treated as a case of Anti-GBM disease potentially triggered by COVID-19. Hence, the anti-GBM disease could be a potential complication of COVID-19.

## Introduction

Severe acute respiratory syndrome coronavirus 2 (SARS-COV-2), the virus causing the COVID-19 pandemic, primarily affects the respiratory tract causing a broad range of respiratory tract infections. It’s true that COVID-19 initially attacks the lungs, but other organs can also be affected, including the kidneys [1]. Kidney injury among hospitalized patients with COVID-19 usually appears during the second week of infection and has a rate ranging from 0.5% to 29% as it appears from the reports of China and Italy [2,3]. The proposed mechanism of kidney injury in patients with COVID-19 is explained by the fact that the kidneys express angiotensin-converting enzyme 2 (ACE2), which is found to be a receptor of the SARS-COV-2 virus, so kidneys could be directly attacked by it [4]. Also, the decrease in oral intake, cytokine storm, and sepsis play a role in kidney injury in these patients [4]. In addition, reports from London showed that the pulmonary-renal syndrome that occurs during the COVID-19 pandemic was in part due to anti-glomerular basement membrane (anti-GBM) disease [5]. Anti-GBM disease -- referred to as anti-GBM syndrome or Goodpasture’s disease -- is a rare small vessel vasculitis. It can affect the capillaries of the glomeruli and cause glomerular necrosis or affect the capillaries of the lung and cause hemorrhage in the alveoli, and sometimes it can affect both of them. This disease is marked by the circulating antibodies that target basement membrane antigens known as Goodpasture antibodies [6]. Recent data showed that the trigger of this disease in vulnerable individuals could be environmental factors including infections [7].

In this article, we report a case of a severe pulmonary-renal syndrome that is admitted to our hospital during the COVID-19 pandemic and was found to have an anti-GBM disease that is most probably triggered by COVID-19. In addition, we highlight the clinical features, diagnosis, treatment of anti-GBM disease, and the possible pathophysiology linking it to the infection.

## Case presentation

A 63-year-old man, an athlete, presented to our emergency department during the COVID-19 pandemic for fever, fatigue, and myalgia. His symptoms started about three weeks before the presentation, and he claimed not to receive any medication or seek medical advice. The patient also reported few episodes of watery diarrhea, however, he denied having cough, chest pain, urinary symptoms, or any recent travel. As for medical history, he is known to have arterial blood hypertension treated with angiotensin receptor blockers (ARB). He does not take any other medication. Surgical and family history are irrelevant. He does not use tobacco or alcoholic products.

Upon presentation, his vital signs were within the normal range. He was afebrile, his blood pressure was 125/85 mmHg, and with oxygen saturation 96% on room air. Chest radiograph revealed bilateral infiltrates. As for the initial workup, complete blood count and basic metabolic profile were ordered. In addition, polymerase chain reaction (PCR) for SARS-CoV 2 was ordered and turned out to be negative. Results on admission showed a normal White blood cell count (WBC) of 4.8/μL with 18% lymphocytes, moderate elevation in erythrocyte sedimentation rate (ESR), and C-reactive protein (CRP) of 33 mm/hr and 31.4 mg/L, respectively. Laboratory values are represented in Table 1.

**Table 1:** Table showing laboratory values on admission, day 10 and day 25 of hospitalization. WBC: white blood cell count; MCV: mean corpuscular volume; ESR: erythrocyte sedimentation rate; BUN: blood urea nitrogen; CRP: C-reactive protein; Ptt: partial thromboplastin time;  Pt: prothrombin time; INR: international normalized ratio; CPK: creatine phosphokinase; C3: complement 3; C4: complement 4; ANCA: anti-neutrophil cytoplasmic antibodies; Anti-GBM: anti-glomerular basement membrane; UA: urine analysis; ABG: arterial blood gas test;  SpO_2_: saturation of oxygen; -: not available

Test		Unit	On admission	Day 10	Day 25
WBC		/μL	4.8	33.6	12.2
Neutrophils		%	72.7	96	92.1
Lymphocytes		%	18	0.9	2.01
Hemoglobin		g/dL	14.4	11.5	12
MCV		fL	86.8	88.7	85.6
Platelet Count		1000/μL	157	226	30
ESR		mm/hr	33	120	-
Albumin		g/dL	4.1	2.5	2.6
Amylase		/L	50	184	-
Lipase		/L	-	90	-
BUN		mg/dL	10	209	160
Creatinine		mg/dL	1	3.5	3.75
Sodium		mmol/L	132	144	139
Potassium		mmol/L	3.5	8.1	5.3
Chloride		mmol/L	101	117	96
Bicarbonate		mmol/L	24	26	25
Total Calcium		mmol/L	9.1	7.2	6
CRP		mg/L	31.4	130.6	64
D-Dimer		ng/mL	6678.33	-	-
Ptt		sec	35.5	35.6	40.1
Pt		sec	13.8	13.7	14.9
INR		-	1.03	1.08	1.13
CPK		-	68	-	46
C3		-	141.8	-	-
C4		-	38.23	-	-
p-ANCA		-	Negative	-	-
c-ANCA		-	Negative	-	-
Anti-GBM		-	Positive	-	-
UA	pH	-	5.5	5.5	5
	gravity	-	1.009	1.012	1.021
	protein	-	Negative	Positive (+)	Positive (+)
	WBC	/μL	0-2	6-10	3-5
	RBC	/μL	0-2	3-5	3-5
	ketone	-	Negative	Negative	Negative
	casts	-	None	None	None
ABG	pH	-	7.43	7.07	-
	P_CO2_	mmHg	29	90.3	-
	P_O2_	mmHg	53	47	-
	HCO3^-^	mmol/L	19.7	26.4	-
	SpO_2_	%	89	63	-

**Figure 1:**
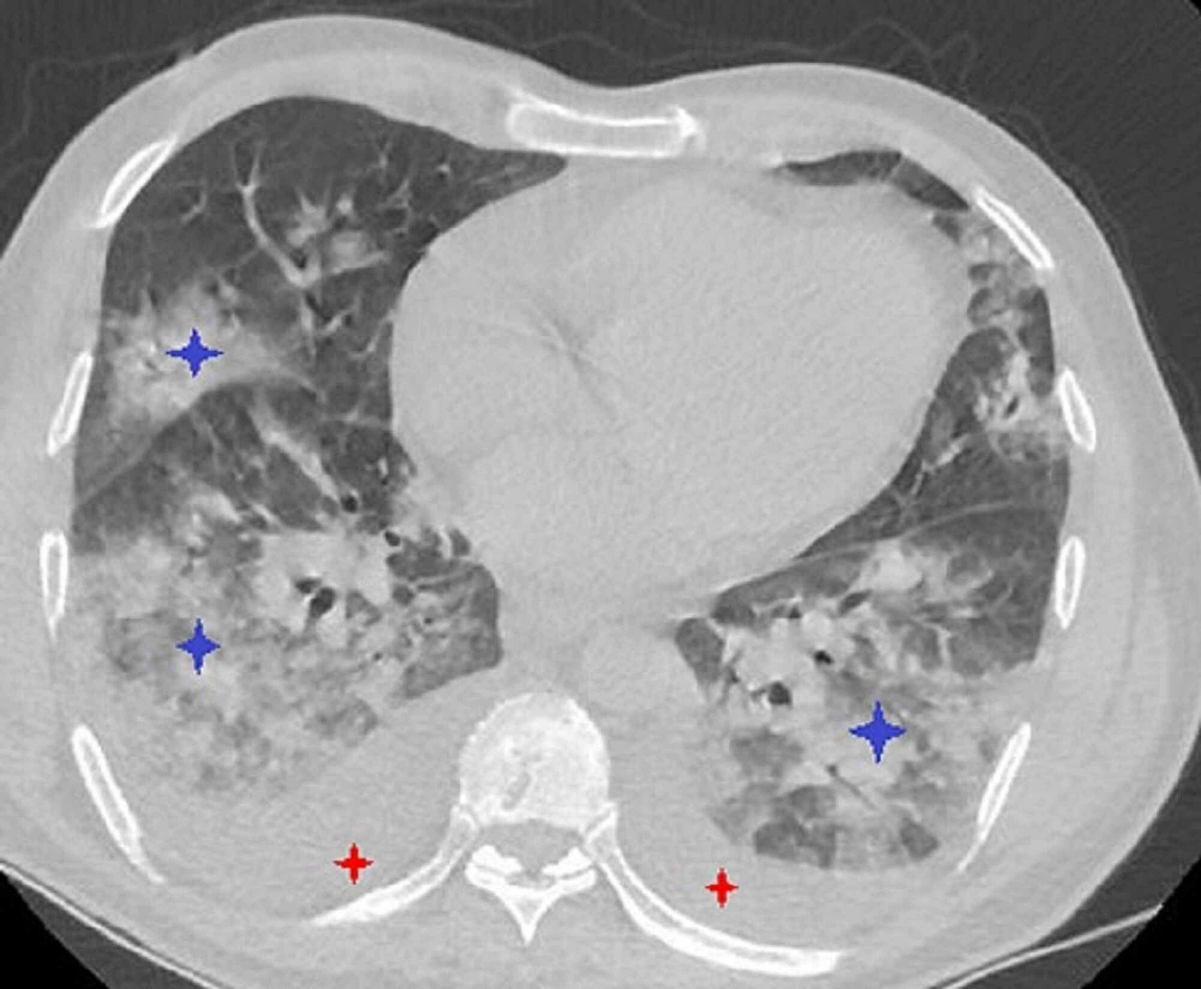
CT of the chest without contrast showing Mild bilateral pleural effusion (red stars); ground-glass opacities with crazy paving aspect predominantly peripheral in distribution (blue stars)

Based on previous results, he was admitted to the regular floor as a case of community-acquired pneumonia and being treated with levofloxacin. Few days after admission, the patient started complaining of increasing dyspnea. Oxygen saturation taken by the oximeter recorded 88% on room air. Thus, oxygen was provided by nasal cannula which improved his saturation, reaching 94%. Repeated lab values revealed an increase in WBC to 15.6/μL, with a decrease in lymphocyte count to 2.9%. ESR was up trending to 80 mm/hr, and renal function started to deteriorate, as revealed by elevated serum creatinine and blood urea nitrogen to 2.9 and 175, respectively. At that point, a urinary catheter was inserted to monitor the urine output, and a CT chest was done to assess his condition, and showed fibrotic bands at the pulmonary bases, bilateral alveolar infiltrates with air-bronchogram, pleural effusion, and ground-glass opacities (Figure 1).

These findings were suggestive of active or previous COVID-19 infection. Thus, a second PCR for SARS-CoV 2 was done, however, with a second negative result. Based on the CT findings and high clinical suspicions, SARS-CoV 2 serology was ordered and turned out to be positive for immunoglobulin M (IgM) and immunoglobulin G (IgG). At this point, we started to treat the patient as a case of COVID-19 infection. He was highly dependent on oxygen without any improvement despite the addition of steroids for management. Therefore, he was transferred to the intensive care unit (ICU). While in ICU the patient developed hemoptysis and a rapid decrease in his glomerular filtration rate along with new-onset proteinuria that was not present upon admission (Table 1). After few days, the patient’s status kept worsening until he developed acute respiratory distress syndrome (ARDS) with oxygen saturation below 85% on a non-rebreather facemask and acute bilateral consolidation seen on chest radiograph. Eventually, ten days after his admission, the patient developed a hypoxic respiratory failure that required urgent intubation. Shortly after that, he became anuric despite being on high doses of diuretics, so hemodialysis was initiated. He was hemodynamically unstable, BP reached 60/45 mmHg requiring inotropes to keep mean arterial pressure above 65 mmHg.

Due to the presence of bilateral lung infiltrates, hemoptysis, new-onset proteinuria, and rapid deterioration of the kidneys function, an autoimmune workup was ordered to rule out autoimmune diseases causing this pulmonary-renal syndrome. Anti-GBM antibody was positive ( 250 EU/mL) whereas the other immune panel (C3, C4, c-ANCA, p-ANCA) turned out to be negative. Due to his low platelet level (less than 50,000/μL) and his critical condition, we did not perform a kidney or lung biopsy. Finally, our diagnosis was anti-GBM syndrome associated with COVID-19. We started him on plasmapheresis and pulse steroid therapy, but yet no improvement, and his status kept worsening despite plasmapheresis, dialysis, and steroids. After few days, the patient had a cardiopulmonary arrest and passed away.

## Discussion

Anti-GBM disease is a rare autoimmune disorder characterized by linear deposition of anti-GBM antibodies, resulting in rapidly progressive glomerulonephritis and pulmonary hemorrhage [8]. The majority of patients present with clinical features of rapidly progressive glomerulonephritis, such as acute kidney injury, macroscopic hematuria, proteinuria, and dysmorphic RBCs in urine analysis [9]. About 40-60% of patients present with both renal manifestations and alveolar hemorrhage, as in our case [6]. A minority of patients have isolated pulmonary findings, including shortness of breath, cough, alveolar hemorrhage that presents as hemoptysis, and infiltrates on chest radiograph [10]. Our case presented with alveolar hemorrhage as compared with recent reports on the co-existence of anti-GBM disease and COVID-19, where patients did not develop alveolar pathology [5]. In addition, our patient deteriorated rapidly compared with a more benign course in other reports [5]. 

The gold standard method for diagnosis of the anti-GBM disease is performing a kidney biopsy. It should be performed in all suspected cases unless there is a contraindication such as thrombocytopenia or hemodynamic instability as in our patient. Serum assay for anti-GBM should be also done using either enzyme-linked immunosorbent assay (ELISA) (sensitivity 94.9% and specificity 97.9%), or indirect immunofluorescence that is rarely used [11]. Treatment is usually provided for all patients with renal involvement, not required urgent dialysis because those who need dialysis are less likely to recover, and for all patients with pulmonary hemorrhage regardless of renal status [12]. Treatment consists of daily plasmapheresis for 2 weeks, then re-measuring the patient's anti-GBM level to determine the need for any further sessions [6]. Patients should also be treated with immunosuppressive therapy that includes steroids and cyclophosphamide. Due to the ongoing infection and the severe condition of our patient, cyclophosphamide was deferred. However, he passed away before initiating cyclophosphamide.

The pathophysiology of the anti-GBM disease occurs at multiple levels: immune, genetic, and environmental. At the immune level, experimental studies have proven a significant role of cell-mediated autoimmunity, particularly the autoreactive T cells [13]. This autoimmune response is directed against the Good-Pasture antigen, which is a non-collagenase domain of the alfa 3 chain of type 4 collagen [a3(IV)NC1] found primary in the glomerular and alveolar basement membrane. This is considered to be the hallmark in the disease pathophysiology [13]. At the genetic level, human leukocyte antigen (HLA) was also found to be strongly associated with anti-GBM disease [6,13]. Moreover, environmental factors precipitating the disease are not fully identified, and the mechanism by which they induce the cascade of events is not established yet [8]. Anecdotally, some trigger factors were considered to be associated with anti-GBM disease, such as smoking, hydrocarbon exposure, lithotripsy, and infection [8]. The exact mechanism by which the infection triggers the disease is yet not well defined. However, it could be explained by the possible role of inflammation in unmasking certain epitopes in the basement membrane permitting access to Goodpasture antibodies [7,8,13].

## Conclusions

In conclusion, our case as well as recently reported cases have shown the possible association between COVID-19 and anti-GBM disease. The mechanism of the development of anti-GBM disease following COVID-19 is not clear yet. Such association is essential to be considered in each case of COVID-19 showing an acute pulmonary deterioration in addition to a decrease in renal function. For that, it is reasonable to request an anti-GBM disease panel facing similar clinical presentations in order not to miss potential life-threatening conditions that have a completely different therapeutic approach.
